# Critical windows of prenatal heat exposure and preterm birth: Metabolomic study in the Atlanta African American Maternal-Child Cohort

**DOI:** 10.1126/sciadv.adw8328

**Published:** 2025-11-21

**Authors:** Kaitlin R. Taibl, Priyanka A. Bhanushali, Anne L. Dunlop, Howard H. Chang, Dana Boyd Barr, Youran Tan, Zhihao Jin, Noah Scovronick, Stephanie M. Eick, P. Barry Ryan, Jeremy A. Sarnat, Carmen J. Marsit, Dean P. Jones, Donghai Liang

**Affiliations:** ^1^ Gangarosa Department of Environmental Health, Rollins School of Public Health, Emory University, Atlanta, GA, USA.; ^2^ Department of Gynecology and Obstetrics, School of Medicine, Emory University, Atlanta, GA, USA.; ^3^ Department of Biostatistics and Bioinformatics, Rollins School of Public Health, Emory University, Atlanta, GA, USA.; ^4^ Division of Pulmonary, Allergy, and Critical Care Medicine, Department of Medicine, School of Medicine, Emory University, Atlanta, GA, USA.

## Abstract

Extreme heat is a risk factor for preterm birth (PTB), but the underlying biological pathways remain poorly understood. This study aimed to identify maternal metabolomic signatures associated with prenatal heat exposure and PTB. We conducted a prospective analysis of 215 participants in the Atlanta African American Maternal-Child Cohort (2014 to 2020). Serum samples collected during early and late pregnancy underwent untargeted metabolomic profiling. Daily maximum ambient temperature was estimated at geocoded residential addresses and averaged over three exposure windows: conception to early pregnancy, early to late pregnancy, and conception to late pregnancy. Metabolome-wide association studies were performed for each exposure window and PTB, followed by a meet-in-the-middle analysis. We identified 23 metabolic pathways and four overlapping metabolites, including methionine, proline, citrulline, and pipecolate, associated with both temperature exposure and PTB. These metabolites are involved in amino acid metabolism and oxidative stress regulation. Findings highlight the potential of metabolomics to detect early biological alterations linked to environmental risk and adverse birth outcomes.

## Teaser

Metabolomic study identified shared biological pathways and metabolites associated with prenatal heat exposure and preterm birth.

## INTRODUCTION

The World Health Organization named climate change as a top threat to population health in the 21st century (*1*). Climate change is also recognized as an environmental injustice issue because certain populations are more vulnerable to its health effects. A growing body of evidence suggests that the gradual increase in ambient temperature from human activity amplifies other environmental hazards among minority races and ethnicities in relation to adverse birth outcomes (*2*–*4*). For example, extreme heat is a leading risk factor for death among infants, preterm birth (PTB; delivery between 22 and 36 gestational weeks), which disproportionately affects African American and Black families in the United States of America (USA) (*5*, *6*). There is an estimated 11.6% increase in the rate of PTB for every 10 F° or 5.6°C increase in ambient temperature between May and October, with stronger associations among Black women and babies (*7*, *8*). Similar observations were made in a retrospective cohort study of 53 million births across 50 metropolitan areas in the USA; non-Hispanic Black women were more likely to deliver preterm from a preceding consecutive heatwave compared to all other racial and ethnic groups (*9*).

Despite few molecular epidemiology studies performed to date, there is biological plausibility that hot weather increases the risk of PTB. Preliminary evidence suggests that the underlying biomechanisms involve oxidative stress (i.e., isoprostanes), inflammation (i.e., angiogenic factors, heat shock proteins, and stress hormones), vasoconstriction in utero, premature rupture of membranes, and onset or exacerbation of cardiometabolic morbidities (*10*, *11*). However, the specific pathways and markers underlying this association remain unexplored to date. Untargeted metabolomics may provide a unique opportunity to characterize such signatures; this high-throughput, high-resolution technique enables the identification and measurement of biological responses from environmental conditions in relation to a health phenotype (*12*, *13*). Several prospective pregnancy cohorts profiled the metabolome for signatures of PTB, including the Atlanta African American Maternal-Child Cohort, and found distinct metabolic patterns relative to early term births (delivery between 37 and 38 gestational weeks) and full term births (delivery after 38 gestational weeks) (*14*–*17*). To our knowledge, only one metabolome-wide association study (MWAS) has been conducted to examine the effects of heat exposure on human metabolome to date, while none focused on prenatal heat exposure and birth outcomes (*18*).

We performed a prospective metabolomic analysis in the Atlanta African American Maternal-Child Cohort, which includes Black residents of the Southeastern metropolitan area with varied socioeconomic status. Our objective was to characterize metabolic pathways and metabolites underlying the association between ambient temperature exposure during pregnancy and PTB. In addition, the overlapping sets of metabolic pathways and metabolites for three exposure periods were compared to identify any critical windows of vulnerability.

## RESULTS

This prospective study included 215 African American who donated serum metabolomics data at two prenatal time points and subsequently delivered a PTB (*N* = 41) or full term birth (*N* = 174) (Table 1). Among these participants, the average maternal age was 25 years, and the average gestational age at birth was 39 weeks. Our study population included 37.7% with obesity, 37.2% with a high school education, 51.6% with a single marital status, and 54.4% with no recent use of alcohol, tobacco, or marijuana. Most conceptions occurred during summer (*N* = 66), followed by spring (*N* = 61), winter (*N* = 48), and then fall (*N* = 40). Last, the mean daily maximum ambient temperature (DMAT) for the three exposure periods by birth term is presented in Table 2.

**Table 1.** Characteristics of pregnant women in the study population, Atlanta African American Maternal-Child Cohort, 2014 to 2020. *N*, number.

	Preterm birth	Full term birth	Overall
*N*	41	174	215
Age (years)	25 (4.3)	25 (4.9)	25 (4.8)
Body mass index (kg/m^2^)			
Underweight (<18.5)	0 (0%)	9 (5.2%)	9 (4.2%)
Normal weight (18.5–24.9)	18 (43.9%)	57 (32.8%)	75 (34.9%)
Overweight (25–29.9)	14 (34.1%)	36 (20.7%)	50 (23.3%)
Obese (≥ 30)	9 (22.0%)	72 (41.4%)	81 (37.7%)
Education		Power	
Less than high school	4 (9.8%)	22 (12.6%)	26 (12.1%)
High school	21 (51.2%)	59 (33.9%)	80 (37.2%)
Some college	13 (31.7%)	56 (32.2%)	69 (32.1%)
College graduate or above	3 (7.3%)	37 (21.3%)	40 (18.6%)
Parity			
0	13 (31.7%)	85 (48.9%)	98 (45.6%)
1	9 (22.0%)	46 (26.4%)	55 (25.6%)
≥2	19 (46.3%)	43 (24.7%)	62 (28.8%)
Marital status			
Married or cohabitating	19 (46.3%)	85 (48.9%)	104 (48.4%)
Single	22 (53.7%)	89 (51.1%)	111 (51.6%)
Use of alcohol, marijuana, or tobacco			
No	18 (43.9%)	99 (56.9%)	117 (54.4%)
Yes	23 (56.1%)	75 (43.1%)	98 (45.6%)
Conception season			
Fall	6 (14.6%)	34 (19.5%)	40 (18.6%)
Spring	15 (36.6%)	46 (26.4%)	61 (28.4%)
Summer	11 (26.8%)	55 (31.6%)	66 (30.7%)
Winter	9 (22.0%)	39 (22.4%)	48 (22.3%)
Infant sex			
Male	29 (70.7%)	79 (45.4%)	108 (50.2%)
Female	12 (29.3%)	95 (54.6%)	107 (49.8%)
Weeks at early pregnancy	12 (2.5)	11 (2.1)	11 (2.2)
Weeks at late pregnancy	26 (2.0)	27 (2.6)	27 (2.5)
Weeks at delivery	35 (2.6)	40 (0.71)	39 (2.4)

**Table 2.** Mean DMAT (°C) for three exposure periods during pregnancy, Atlanta African American Maternal-Child Cohort, 2014 to 2020 (*N* = 215). Note: Reported as mean (SD).

	Preterm birth	Full term birth	Overall
*N*	41	174	215
Conception–early pregnancy (0–17 gestational weeks)	27 (7.0)	26 (7.3)	26 (7.2)
Early pregnancy–late pregnancy (21–35 gestational weeks)	25 (6.6)	25 (6.4)	25 (6.4)
Conception–late pregnancy (0–35 gestational weeks)	26 (4.7)	25 (4.6)	25 (4.6)

### Molecular signatures in maternal metabolome-wide association studies

We extracted 13,616 and 11,900 metabolic features from the hydrophilic interaction liquid chromatography (HILIC)–positive electrospray ionization (ESI) column and C18-negative ESI column, respectively. These features were used to conduct the 10 MWAS (table S1). At *P* < 0.05, ambient temperature exposure during conception to late pregnancy was associated with the greatest number of metabolic features (HILIC = 1103; C18 = 673). For the MWAS corresponding to conception to early pregnancy exposure, there were 662 and 484 features yielded by the HILIC and C18 columns, respectively. For the MWAS corresponding to early pregnancy to late pregnancy exposure, there were 691 and 489 features yielded by the HILIC and C18 columns, respectively. Last, metabolomic profiling of maternal serum from either visit revealed that PTB was associated with more features in the HILIC column (early pregnancy: 683; late pregnancy: 636) than the C18 column (early pregnancy: 585; late pregnancy: 535).

The number of metabolic pathways enriched in each MWAS is presented in table S2. We found 14, 22, and 20 metabolic pathways in the HILIC MWAS for the exposure period corresponding to conception to early pregnancy, conception to late pregnancy, and early pregnancy to late pregnancy, respectively. We also found 19, 19, and 17 metabolic pathways in the C18 MWAS for the exposure period corresponding to conception to early pregnancy, conception to late pregnancy, and early pregnancy to late pregnancy, respectively. At early pregnancy, PTB was associated with 17 metabolic pathways in the HILIC column and 18 metabolic pathways in the C18 column. In comparison, at late pregnancy, PTB was associated with 27 metabolic pathways in the HILIC column and 13 metabolic pathways in the C18 column.

### Overlapping metabolomic signatures from meet-in-the-middle analysis

**Fig. 1.**
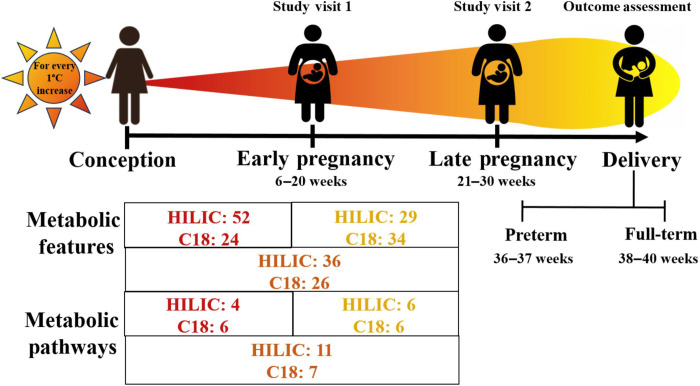
Metabolomic signatures for the association between ambient temperature exposure and PTB in the maternal metabolome, Atlanta African American Maternal-Child Cohort, 2014 to 2020 (*N* = 215). Models adjusted for conception season, maternal age, education, body mass index (BMI), parity, recent use of alcohol, marijuana, or tobacco, gestational age at sample collection, and infant sex. C18, reverse phase chromatography column.

**Fig. 2.**
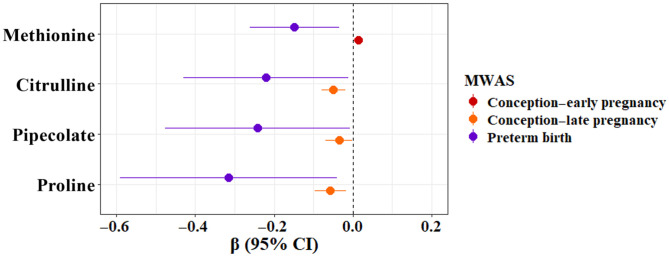
Forest plot of overlapping metabolites confirmed with level-1 confidence for the association between ambient temperature exposure and PTB in the maternal metabolome, Atlanta African American Maternal-Child Cohort, 2014 to 2020 (*N* = 215). All metabolites enriched in the HILIC column. Models adjusted for conception season, maternal age, education, BMI, parity, recent use of alcohol, marijuana, or tobacco, gestational age at sample collection, and infant sex. Red point estimate and CI denote the association between ambient temperature exposure during conception to early pregnancy time period and the intermediate metabolite methionine in the maternal metabolome with serum collected at early pregnancy. Orange point estimate and CI denote the association between ambient temperature exposure during conception to late pregnancy time period and the intermediate metabolites citrulline, pipecolate, and proline in the maternal metabolome with serum collected at late pregnancy. Purple point estimate and CI denote the association between PTB (referent: full term birth) and the intermediate metabolites methionine, citrulline, pipecolate, and proline in the maternal metabolome with serum collected at early pregnancy.

We identified metabolic features that overlap with ambient temperature during any of the three exposure periods and PTB before confirmation and annotation (Fig. 1). Four metabolites were confirmed with level-1 confidence in the HILIC column (Fig. 2 and table S3). In particular, methionine was positively associated with the conception to early pregnancy exposure period [β = 0.01, 95% confidence interval (CI) = 0.001 and 0.03] and negatively associated with PTB (β = −0.15, 95% CI = −0.26 and −0.03). All other metabolites had a negative association with the conception to late pregnancy exposure period and PTB. Specifically, for every 1°C increase in ambient temperature during conception to late pregnancy, there was a 0.06 (95% CI = −0.10 and −0.02) and 0.04 (95% CI = −0.07 and −0.001) decrease in the log_2_ intensity of proline and pipecolate, respectively. Both metabolites had a negative effect on PTB (proline: β = −0.32, 95% CI = −0.59 and −0.04; pipecolate: β = −0.24, 95% CI = −0.48 and −0.01). Last, citrulline was negatively associated with the conception to late pregnancy exposure period (β = −0.05, 95% CI = −0.08 and −0.02) and PTB (β = −0.22, 95% CI = −0.43 and −0.01).

**Fig. 3.**
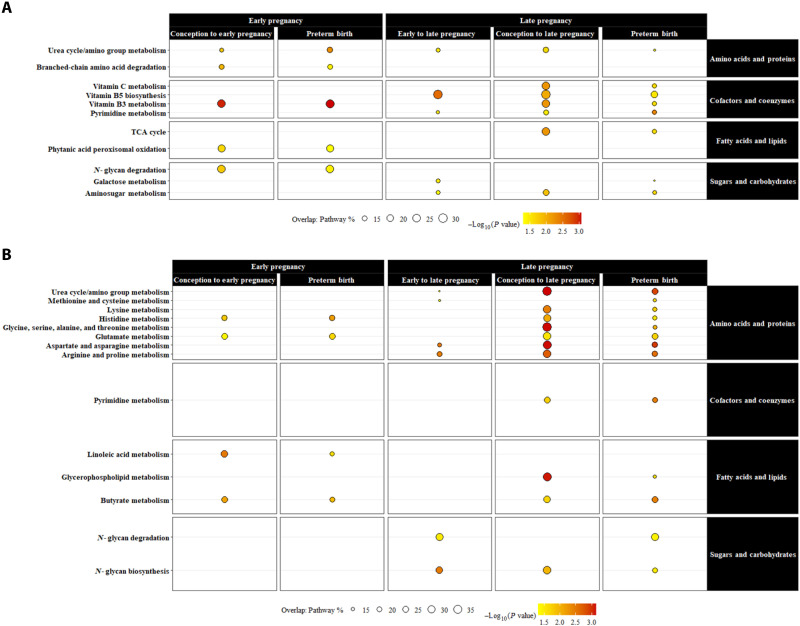
Overlapping metabolic pathways associated with ambient temperature exposure and PTB in the maternal metabolome, Atlanta African American Maternal-Child Cohort, 2014 to 2020 (*N* = 215). (**A**) C18. (**B**) HILIC. Pathways were classified by study visit (early pregnancy, first or late pregnancy, or second) and exposure window (conception to early pregnancy, early pregnancy to late pregnancy, and conception to late pregnancy), and adverse birth outcome was matched to exposure periods according to the timing of sample collection. Furthermore, pathways were grouped by function including amino acids and proteins, cofactors and coenzymes, fatty acids and lipids, and sugars and carbohydrates. Pathways were designated as “intermediate” if they overlapped with ambient temperature during any of the three exposure windows and PTB within the same study visit for sample collection.

The metabolic pathways that overlap with ambient temperature during any of the three exposure periods and PTB are shown in Fig. 3 and table S4. Urea cycle/amino group metabolism was enriched in all MWAS. Only in the HILIC column, linoleic acid metabolism overlapped with the conception to early pregnancy exposure period and PTB, methionine and cysteine metabolism and *N*-glycan degradation overlapped with the early pregnancy to late pregnancy exposure period and PTB, and metabolism of lysine, glycine, serine, alanine, threonine, pyrimidine, and glycerophospholipid overlapped with the conception to late pregnancy exposure period and PTB. Metabolic pathways for histidine, glutamate, and butyrate were enriched in the HILIC MWAS for the conception to early pregnancy exposure period, conception to late pregnancy exposure period, and PTB. Last, metabolic pathways for aspartate, asparagine, arginine, and proline plus *N*-glycan biosynthesis were enriched in the HILIC MWAS for the early pregnancy to late pregnancy exposure period, conception to late pregnancy exposure period, and PTB. Only in the C18 column, branched-chain amino acid degradation, *N*-glycan degradation, and phytanic acid peroxisomal oxidation overlapped with the conception to early pregnancy exposure period and PTB, galactose metabolism overlapped with the early pregnancy to late pregnancy exposure period and PTB, and vitamin C metabolism and the citric acid (TCA) cycle overlapped with the conception to late pregnancy exposure period and PTB. The metabolic pathway for vitamin B3 was enriched in the C18 MWAS for the conception to early pregnancy exposure period, conception to late pregnancy exposure period, and PTB. Last, pathways for vitamin B5 biosynthesis and aminosugar metabolism were enriched in the C18 MWAS for the early pregnancy to late pregnancy exposure period, conception to late pregnancy exposure period, and PTB.

### Sensitivity analyses

The association between ambient temperature during pregnancy and PTB was null (table S5). We also reported the mean DMAT for the acute exposure periods in table S6. For ambient temperature exposure during the 1 week before early pregnancy to early pregnancy and the 1 week before late pregnancy to late pregnancy, there tended to be fewer metabolic features (table S7) and metabolic pathways (table S8) than those observed in the main analysis for chronic exposure periods.

## DISCUSSION

**Fig. 4.**
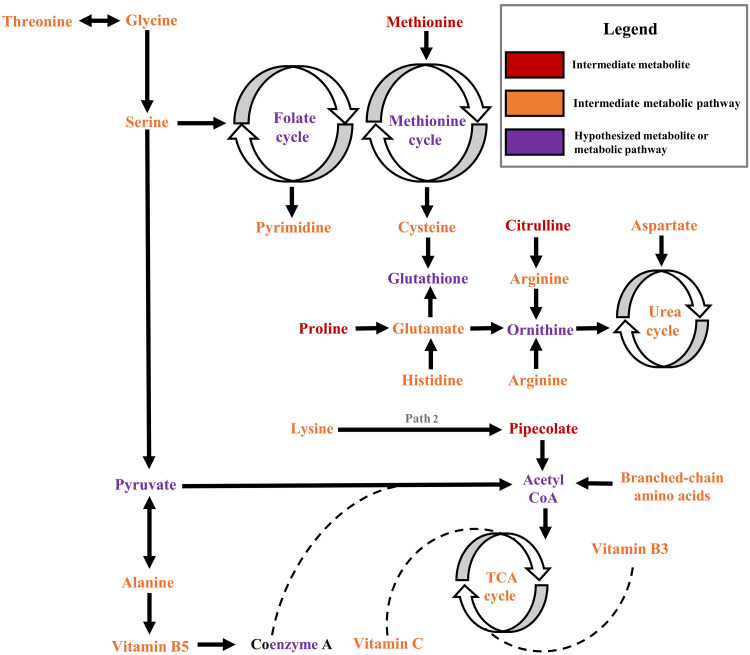
Hypothesized diagram of amino acids and vitamins underlying the association between ambient temperature exposure and PTB in the maternal metabolome, Atlanta African American Maternal-Child Cohort, 2014 to 2020.

In this prospective analysis of pregnant African Americans, we characterized several metabolic pathways and metabolites that link higher ambient temperature exposure during pregnancy to higher likelihood of PTB. As shown in our hypothesized mechanistic diagram, amino acid and vitamin perturbations were consistent and strong signatures in the maternal metabolome (Fig. 4). At the mother’s residence, every 1°C increase in the mean DMAT between conception to early pregnancy was associated with higher levels of methionine, while every 1°C increase in the mean DMAT between conception to late pregnancy was associated with lower levels of citrulline, pipecolate, and proline. All four metabolites were lower among participants who delivered before 37 gestational weeks, compared to full term births. In addition, metabolic pathways for the urea cycle and vitamins B3, B5, and C were enriched in the exposure and outcome MWAS. We found evidence of differential metabolomic signatures across exposure windows, suggesting that ambient heat exposure may influence distinct biological pathways in early versus late pregnancy. These findings point to the potential for temporally specific vulnerability, although further studies are needed to confirm whether particular gestational windows represent critical periods for heat-related PTB risk.

A few existing studies suggest that heat exposure leads to preterm labor and delivery via oxidative stress and inflammation (*5*, *10*). Our findings provide additional evidence for the role of these pathophysiological processes and contribute previously unidentified insights about redox dyshomeostasis. In particular, in both pathway enrichment and metabolite confirmation, we identified an essential amino acid, methionine, which is an indirect precursor to glutathione (i.e., peptide of cysteine, glutamate, and glycine). This antioxidant protects against nonenzymatic oxidative stress, the pathway for lipid peroxidation, a signature of thermal stress, and PTB (*19*, *20*). Proline, another amino acid pathway and metabolite we identified as a signature in the maternal metabolome, is important for maintaining the glutathione pool and scavenging reactive oxygen species (*21*). Likewise, an experimental investigation of female chickens found that proline mitigates thermal stress through glutathione production and heat shock protein activation (*22*). Proline also undergoes interconversion with glutamate to enter the TCA cycle, which is supported by vitamins B3, B5, and C, and provides a predominant source of energy for the mother, placenta, and fetus during pregnancy. Pathways for biosynthesis, metabolism, and degradation of these vital amino acids and vitamins, plus the TCA cycle, were enriched in our study of the maternal metabolome. However, low proline has been reported among PTBs in previous work and the present study, which may indicate impaired mitochondrial bioenergetics (*23*).

The mitochondrial matrix is where the TCA cycle and start of the urea cycle occur. For the latter metabolic pathway, which was strongly and consistently associated with the exposure and outcome here, the liver converts ammonia, a toxic by-product of amino acid metabolism, to urea, before the kidneys excrete in urine. Both organs are prone to tissue damage from thermal stress and more so among pregnant women, who have higher body temperatures than other adults, and fetuses, which cannot regulate body temperatures until the third trimester (*24*). Furthermore, the liver and kidneys contain citrulline for synthesis of urea and nitric oxide from arginine, respectively. The bioavailability of nitric oxide, a vasodilator, increases in hot environments and decreases in preterm deliveries, as evidenced by prior work (*25*, *26*). Our finding of the arginine and proline metabolic pathway and citrulline metabolite adds to this knowledge, indicating that chronically high ambient temperatures may overwhelm metabolic pathways or deplete metabolites for nitric oxide synthesis during pregnancy.

The final signature we identified in the maternal metabolome was pipecolate (i.e., pipecolic acid), a metabolite of the second pathway of lysine degradation in liver peroxisomes. Not all steps of pipecolate formation have been elucidated but possible explanations for our finding include lysine deficiency or lysine degradation via the first pathway in the mitochondria for the supply of acetyl coenzyme A (CoA) in the TCA cycle (*27*). This essential amino acid enhances calcium absorption and urination, two processes that increase during pregnancy but decrease in hot environments. The identification of pipecolate in other PTB studies warrants follow-up investigation for the metabolic connection to prenatal heat exposure (*28*–*30*). Some metabolites demonstrated opposite directions of association with temperature exposure and PTB risk, reflecting the complexity of pregnancy-related metabolic responses. These patterns may indicate compensatory or adaptive mechanisms to heat stress that protect against adverse outcomes, while others may reflect maladaptive pathways contributing to PTB. These findings highlight the importance of pathway-level interpretation and underscore the need for mechanistic follow-up studies to disentangle the dual roles that some metabolites may play in environmental adaptation and disease processes.

### Strengths and limitations

A strength of this original meet-in-the-middle analysis of the maternal metabolome that links ambient temperature exposure during pregnancy to PTB was our focus on the African American study population. Climate change poses a higher risk to vulnerable groups such as pregnant women and racial or ethnic minorities, who are represented in the Atlanta African American Maternal-Child Cohort. We geocoded the mothers’ residential addresses at early pregnancy to link with the maximum ambient temperature for days between conception and delivery. This advantage was enhanced by the repeated collection of maternal serum for high-resolution metabolomics, enabling us to explore critical windows of vulnerability for heat exposure. Last, we refer to “windows of vulnerability” rather than “windows of susceptibility” to acknowledge the broader context in which environmental exposures may lead to adverse outcomes. Whereas susceptibility captures biological sensitivity, vulnerability additionally accounts for differential exposure patterns and the capacity for adaptive response—dimensions that are particularly important when studying historically marginalized populations at disproportionate risk for both environmental exposures and adverse birth outcomes (*31*).

Our study focused on evaluating the risk of PTB among our cohort participants whose pregnancy ended in live birth (delivery on or after 22 gestational weeks). However, a growing number of studies support an association between high ambient temperature and risk of impaired fecundity and spontaneous abortion (*32*). Although we did not observe a statistically significant association between DMAT and PTB, our meet-in-the-middle analysis (*16*, *33*) was designed to explore potential metabolic signatures, which bridge exposure and outcome. Unlike formal mediation models, this approach does not require a significant total effect and is particularly suited for identifying biologically plausible intermediates in complex exposure-outcome relationships. Larger cohorts with ample power for detecting the rate of PTB in the overall study population are needed to fully capture the spectrum of heat-related pregnancy outcomes. Furthermore, although we adjusted for infant sex, maternal body mass index (BMI), and parity in all metabolomic models, residual confounding cannot be entirely ruled out. Future studies with larger sample sizes may benefit from stratified analyses or formal interaction testing to explore whether these factors also modify the association between ambient temperature exposure, maternal metabolism, and PTB risk. In addition, because of lack of sufficient power, we did not adjust for air pollution exposures, which often co-occur with elevated temperatures and may confound the observed associations with PTB and metabolomic signatures (*17*, *34*, *35*). Future studies with access to both temperature and air quality data should consider joint modeling of these exposures to disentangle their independent and combined effects.

Other limitations included the lack of participant data on time spent outdoors and temperature indoors plus lack of serum samples for the immediate days before delivery. The latter exposure scenario has been reported as the most influential period by other investigations. Because of the lack of serum samples collected in the final days before delivery, we were unable to assess acute temperature exposures in the immediate predelivery period. Future studies with more frequent sampling near the time of delivery may better characterize the role of short-term heat exposure in PTB risk. While our study focused on average ambient temperature exposures over extended gestational windows, we acknowledge that short-term or acute temperature fluctuations in the days or weeks leading up to delivery may also influence the risk of PTB. Prior studies have identified associations between short-term heat exposure and PTB (*36*), potentially through mechanisms distinct from those linked to cumulative gestational exposure. However, because of limitations in cohort size and exposure frequency, our study was not powered to evaluate specific lag structures or acute temperature thresholds. Future investigations with larger sample sizes and higher temporal resolution are needed to fully characterize the contributions of both short- and long-term temperature exposures to adverse pregnancy outcomes. Last, we did not correct for the multiple comparisons in the metabolome-wide association analysis (*31*, *37*). Although false discovery rate (FDR) correction reduces the likelihood of false positives, it may also increase the risk of false negatives—potentially excluding biologically meaningful signals, especially in studies with modest sample sizes. Hence, we focused our interpretation on consistent patterns and conducted pathway enrichment analysis using metabolic features, meeting a nominal *P* < 0.05. While environmental epidemiologic studies do not commonly adjust for multiple comparisons because of the modest sample sizes, there is an increased probability of false positives, particularly for high-dimensional omics analyses. Hence, the findings should be considered exploratory and hypothesis generating, requiring confirmation in larger, independent cohorts. Last, although preliminary, the metabolomic pathways identified in this study—such as those involved in antioxidant defense, mitochondrial function, and one-carbon metabolism—could help generate hypotheses for targeted nutritional or behavioral interventions. For example, micronutrient supplementation strategies supporting metabolic resilience during periods of thermal stress may warrant investigation in future studies. Larger, longitudinal cohorts will be critical for evaluating whether such pathways are modifiable and causally linked to improved birth outcomes.

### Conclusion and outlook

In a prospective analysis of 215 African American pregnant women living in metropolitan Atlanta, we characterized metabolomic signatures for the association between ambient temperature exposure and PTB. Overall, we identified critical perturbations in maternal serum amino acids and vitamins that support redox homeostasis. A critical window of vulnerability for heat exposure was not observed but most overlapping metabolites and metabolic pathways were associated with exposure spanning from conception to late pregnancy. This work demonstrates the potential utility of metabolomics to obtain molecular insights that may contribute to future development of nutritional interventions that protect maternal-child health from thermal stress, especially in the context of climate change.

## MATERIALS AND METHODS

### Atlanta African American Maternal-Child Cohort design and study population

Participants in the present analysis were those first consecutively enrolled in the prospective Atlanta African American Maternal-Child Cohort, which has the overall goal to improve understanding about health effects of environmental exposures in early life (*38*, *39*). Our study population included women with two prenatal serum samples for metabolomic analysis and whose pregnancy ended in a PTB or full term birth (fig. S1). We excluded miscarriages, abortions, and stillbirths, as the pathophysiology of such outcomes may be different than that for live births. We also excluded participants who visited only once because an objective was to study windows of vulnerability, which required at least two time points. Recruitment took place at Emory University Hospital Midtown and Grady Memorial Hospital in metropolitan Atlanta, Georgia, USA, between 2014 and 2020. Women were eligible to participate if they (i) were pregnant with a singleton in the first or second trimester, (ii) self-identified race as African American or Black, (iii) born in the US, (iv) aged 18 to 40 years, (v) proficient in English, and (vi) had no chronic medical conditions. All participants provided written, informed consent. This study was approved by the Institutional Review Board at Emory University (#68441).

### Anthropometric, sociodemographic, and clinical data ascertainment

At the early pregnancy visit (range = 6 to 17 gestational weeks, mean = 11 gestational weeks), sociodemographic data (i.e., maternal age, education, medical insurance type, and marital status) and use of alcohol, tobacco, or marijuana in the past month were ascertained by standardized interview surveys; anthropometric data (i.e., maternal height and weight) were abstracted from medical records to calculate BMI (in kilograms per square meter); and venous blood samples were collected. At the late pregnancy visit (range = 21 to 35 gestational weeks, mean = 27 gestational weeks), venous blood samples were collected again.

During pregnancy and after delivery, medical records were abstracted by trained staff to ascertain parity, infant sex, and gestational age at birth. The best obstetrical estimate was based on the delivery date and estimated date of confinement, ultrasound, and/or last menstrual period at the early pregnancy visit (*40*). Birth terms were categorized according to gestational age windows (*41*). The estimated date of confinement was also used to calculate conception season (winter = 21 December to 20 March, spring = 21 March to 20 June, summer = 21 June to 20 September, and fall = 21 September to 20 December), which reflects seasonal patterns in the metropolitan Atlanta area.

### Ambient temperature exposure assessment

Ambient temperature data were collected from Daymet, a publicly available database supported by the NASA (*42*). In the US, daily meteorological observations are used to estimate gridded patterns of weather with 1 km–by–1 km spatial resolution. We matched the DMAT exposure (in °C) to each date during pregnancy (conception to delivery) based on the geocoded residential address of the mother at early pregnancy. For all participants, we averaged the DMAT from conception to early pregnancy, early pregnancy to late pregnancy, and conception to late pregnancy. These exposure periods were selected to enable comparison of two potential windows of vulnerability and across pregnancy. The maximum ambient temperature for days after the visit at late pregnancy and before delivery was discarded to maintain temporality among the exposure, metabolome, and outcome in subsequent analyses.

### Untargeted high-resolution metabolomics

Venous blood samples were collected from nonfasting participants, centrifuged for serum extraction, and then stored at −80°C before analysis by the Emory Clinical Biomarker Laboratory (*43*–*45*). We used an established, untargeted workflow to conduct high-resolution metabolomic profiling on maternal serum samples (*46*, *47*). To enhance the coverage of metabolic features for each biosample, two chromatography columns were used, including HILIC with positive ESI and reversed phase (C18) chromatography with negative ESI. Liquid chromatography–high-resolution mass spectrometry was operated in full-scan mode at 120,000 resolution to cover a range of mass/charge ratio (*m/z*) from 85 to 1275. Two internal standards, including pooled serum and standard reference material for human metabolites in plasma (NIST SRM 1950), were added at the beginning and end of each batch with 20 biosamples for quality control, batch correction, and standardization (*48*). The raw instrument files were converted to mzML format, and the resultant metabolic features were extracted and aligned using apLCMS with modification of xMSanalyzer (*49*, *50*).

Before statistical analyses, additional quality control measures were performed to optimize the data quality. Signal noise was filtered out by the exclusion of technical replicates with a coefficient of variation > 30% and Pearson correlation coefficient < 0.70 for metabolic features detected in less than 15% of biosamples. Intensities of the remaining metabolic features were averaged across triplicates and normalized by log_2_ transformation. All metabolomic analyses were performed using established quality assurance and quality control protocols (*46*, *51*). Two internal standards, which include pooled serum and standard reference material for human metabolites in plasma (NIST SRM 1950), were added at the beginning and the end of each batch of 20 samples for quality control and standardization. Instrument blanks and process blanks were included to detect potential contamination. After instrument analysis (*49*, *52*), raw instrument files were converted to .mzML, and metabolic signals were extracted and aligned by apLCMS with modification of xMSanalyzer, which enhanced data quality control and reduced batch effects. To filter out the noise signals and optimize the metabolomic data quality, we excluded the metabolic features, which were detected in <15% of the samples, with coefficient of variation among technical replicates > 30%, and with Pearson correlation coefficient < 0.7.

### Statistical analysis

We used multiple linear regression to model the effect of mean daily maximum temperature for the three exposure periods (conception to early pregnancy, early pregnancy to late pregnancy, and conception to late pregnancy) and PTB (referent: full term birth) onto the log_2_-transformed intensity of each detected metabolic feature. To avoid temporal bias, the end time for each exposure period was matched to the gestational age at biosample collection. Specifically, we analyzed exposure during conception to early pregnancy with PTB in the early pregnancy metabolome and exposure during early pregnancy to late pregnancy or conception to late pregnancy with PTB in the late pregnancy metabolome. Last, all MWASs were performed with features detected in the C18 and HILIC chromatography columns, which totaled 10 MWAS.

Seasonality of conception can confound the association between seasonal-varying exposures, such as ambient temperature, and birth outcomes (*53*–*55*). To minimize bias from conception season, we included this confounder in all models. Other covariates included maternal age, education, BMI, parity, use of alcohol, marijuana, or tobacco in the month before early pregnancy, gestational age at sample collection, and infant sex. We followed the Benjamini-Hochberg procedure to correct for multiple comparisons and used R (Boston, MA, USA; version 4.4.0) to perform all statistical analyses (*56*).

### Pathway enrichment analysis

We used mummichog, an innovative bioinformatics tool, to predict biological function of significant metabolic features (*57*). A limited number of significant features were identified after FDR correction with the Benjamini-Hochberg procedure (table S9). Given the hypothesis generation nature of this study, we opted to use an unadjusted *P* < 0.05 as the significance threshold to ensure enough features for the pathway enrichment analyses. While multiple-testing correction restricts the possibility of false positives, this procedure may also filter out weaker but still relevant features, particularly due to the intercorrelated nature of metabolomic data.

### Metabolite confirmation and annotation

The extracted ion chromatograph for each significant metabolic feature was examined for clear Gaussian peak shapes and signal-to-noise ratios above 3:1 to reduce type II error. Metabolic features that exhibited clear, differentiated peaks were then annotated and confirmed with the Metabolomics Standards Initiative criteria; level-1 confidence was assigned to those with a difference of *m/z* ± 10 parts per million and retention time ± 30 s from matched authentic compounds analyzed under identical experimental conditions (*58*).

### Meet-in-the-middle analysis

The overlapping metabolic pathways and metabolites for the association between ambient temperature exposure and PTB were identified with the meet-in-the-middle framework (*59*–*61*). Specifically, metabolic pathways and metabolites associated with an exposure period and outcome MWAS, plus matched on chromatography column, were considered overlapping metabolomic signatures.

### Sensitivity analysis

In addition to the main analysis of chronic ambient temperature exposure during pregnancy, we examined acute ambient temperature exposure during the 1 week before early pregnancy to early pregnancy and 1 week before late pregnancy to late pregnancy. In another sensitivity analysis, we estimated the associations between chronic exposure periods during pregnancy and PTB.
