# Supplementary material for: Critical windows of prenatal heat exposure and preterm birth: Metabolomic study in the Atlanta African American Maternal-Child Cohort
Source: Sci Adv. 2025 Nov 21;11(47):eadw8328. doi: 10.1126/sciadv.adw8328 (PMC12637296; doi:10.1126/sciadv.adw8328)
Supplement: Supplementary file 1 — Fig. S1 Tables S1 to S9 [file sciadv.adw8328_sm.pdf]

Science Advances
AAAS

Supplementary Materials for

Critical windows of prenatal heat exposure and preterm birth: Metabolomic study in the Atlanta African American Maternal-Child Cohort

Kaitlin R. Taibl *et al.*

Corresponding author: Donghai Liang, donghai.liang@emory.edu

Sci. Adv. **11**, eadw8328 (2025)
DOI: 10.1126/sciadv.adw8328

This PDF file includes:

Fig. S1
Tables S1 to S9

Figure S1. Participant flowchart of study population drawn from Atlanta African American Maternal-Child Cohort, 2014 - 2020.

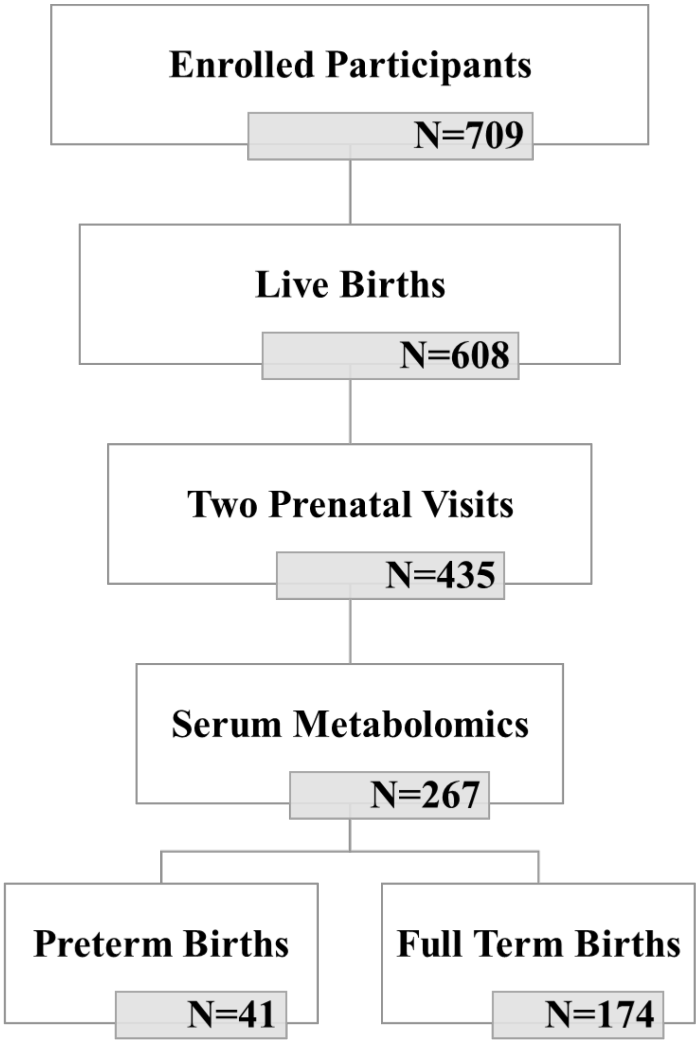

Table S1. Metabolic features associated with ambient temperature exposure and preterm birth in the maternal metabolome, Atlanta African American Maternal-Child Cohort, 2014 - 2020 (N = 215).

Column	Metabolome	Exposure Period	p-value < 0.05
HILIC	Early Pregnancy	Conception – Early Pregnancy	52
	Late Pregnancy	Early Pregnancy – Late Pregnancy	29
		Conception – Late Pregnancy	36
C18	Early Pregnancy	Conception – Early Pregnancy	24
	Late Pregnancy	Early Pregnancy – Late Pregnancy	34
		Conception – Late Pregnancy	26

Abbreviations: C18: reverse phase chromatography column, HILIC: hydrophilic interaction liquid chromatography column.

Table S2. Metabolic pathways enriched in the maternal metabolome, Atlanta African American Maternal-Child Cohort, 2014 - 2020.

Column	Metabolome	Exposure Period	p-value < 0.05
HILIC	Early Pregnancy	Conception – Early Pregnancy	14
		Preterm Birth	17
	Late Pregnancy	Early Pregnancy – Late Pregnancy	20
		Conception – Late Pregnancy	22
		Preterm Birth	27
C18	Early Pregnancy	Conception – Early Pregnancy	19
		Preterm Birth	18
	Late Pregnancy	Early Pregnancy – Late Pregnancy	17
		Conception – Late Pregnancy	19
		Preterm Birth	13

Abbreviations: C18: reverse phase chromatography column, HILIC: hydrophilic interaction liquid chromatography column.

Table S3. Metabolites confirmed with Level-1 confidence associated with ambient temperature exposure and preterm birth in the maternal metabolome, Atlanta African American Maternal-Child Cohort, 2014 - 2020 (N = 215).

Metabolite	m/z	RT	Adduct	Column	MWAS	β	95% CI	p-value
Methionine	150.0583	53.0	M+H	HILIC	Conception – Early Pregnancy	0.01	0.001, 0.03	0.04
					Preterm Birth	-0.15	-0.26, -0.03	0.01
Proline	116.0706	65.0	M+H	HILIC	Conception – Late Pregnancy	-0.06	-0.10, -0.02	0.01
					Preterm Birth	-0.32	-0.59, -0.04	0.03
Pipecolate	130.0868	60.6	M+H	HILIC	Conception – Late Pregnancy	-0.04	-0.07, -0.001	0.04
					Preterm Birth	-0.24	-0.48, -0.01	0.04
Citrulline	176.1035	91.2	M+H	HILIC	Conception – Late Pregnancy	-0.05	-0.08, -0.02	0.002
					Preterm Birth	-0.22	-0.43, -0.01	0.04

Abbreviations: C18: reverse phase chromatography column, HILIC: hydrophilic interaction liquid chromatography column, m/z: mass-to-charge ratio, RT: retention time, MWAS: metabolome-wide association study, CI: confidence interval.

Table S4. Metabolic pathways associated with ambient temperature exposure and preterm birth in the maternal metabolome, Atlanta African American Maternal-Child Cohort, 2014 - 2020 (N = 215).

Pathway	Enrichment Size	Column	Metabolome	Exposure Period	Overlap %	p-value
Urea cycle/amino group metabolism	9	C18	Early Pregnancy	Conception – Early Pregnancy	12.8	0.016
		C18	Early Pregnancy	Preterm Birth	17.0	0.005
		C18	Late Pregnancy	Conception – Late Pregnancy	17.0	0.023
		C18	Late Pregnancy	Early Pregnancy – Late Pregnancy	12.8	0.030
		C18	Late Pregnancy	Preterm Birth	10.6	0.035
		HILIC	Late Pregnancy	Conception – Late Pregnancy	39.1	0.001
		HILIC	Late Pregnancy	Early Pregnancy – Late Pregnancy	13.0	0.049
		HILIC	Late Pregnancy	Preterm Birth	23.9	0.001
Pyrimidine metabolism	7	C18	Late Pregnancy	Conception – Late Pregnancy	16.1	0.033
		C18	Late Pregnancy	Early Pregnancy – Late Pregnancy	12.5	0.028
		C18	Late Pregnancy	Preterm Birth	14.3	0.004
		HILIC	Late Pregnancy	Conception – Late Pregnancy	24.5	0.018
		HILIC	Late Pregnancy	Preterm Birth	18.9	0.004
Aminosugars metabolism	6	C18	Late Pregnancy	Conception – Late Pregnancy	19.4	0.014
		C18	Late Pregnancy	Early Pregnancy – Late Pregnancy	12.9	0.049
		C18	Late Pregnancy	Preterm Birth	12.9	0.021
Vitamin B3 metabolism	6	C18	Early Pregnancy	Conception – Early Pregnancy	23.8	0.001
		C18	Early Pregnancy	Preterm Birth	28.6	0.001
		C18	Late Pregnancy	Conception – Late Pregnancy	23.8	0.006
		C18	Late Pregnancy	Preterm Birth	14.3	0.025
Butyrate metabolism	5	HILIC	Early Pregnancy	Preterm Birth	19.2	0.012
		HILIC	Late Pregnancy	Conception – Late Pregnancy	26.9	0.020
		HILIC	Late Pregnancy	Preterm Birth	23.1	0.004
		HILIC	Early Pregnancy	Conception – Early Pregnancy	23.1	0.008
Glycerophospholipid metabolism	5	HILIC	Late Pregnancy	Conception – Late Pregnancy	37.0	0.001
		HILIC	Late Pregnancy	Preterm Birth	15.2	0.024
Glycine, serine, alanine, and threonine metabolism	5	HILIC	Late Pregnancy	Conception – Late Pregnancy	38.3	0.001
		HILIC	Late Pregnancy	Preterm Birth	17.0	0.009
Lysine metabolism	5	HILIC	Late Pregnancy	Conception – Late Pregnancy	32.1	0.004
		HILIC	Late Pregnancy	Preterm Birth	17.9	0.017
Arginine and proline metabolism	4	HILIC	Late Pregnancy	Conception – Late Pregnancy	32.4	0.002
		HILIC	Late Pregnancy	Early Pregnancy – Late Pregnancy	18.9	0.004
		HILIC	Late Pregnancy	Preterm Birth	21.6	0.003
Vitamin C metabolism	4	C18	Late Pregnancy	Conception – Late Pregnancy	23.8	0.006
		C18	Late Pregnancy	Preterm Birth	14.3	0.025
Aspartate and asparagine metabolism	4	HILIC	Late Pregnancy	Conception – Late Pregnancy	34.8	0.001
		HILIC	Late Pregnancy	Early Pregnancy – Late Pregnancy	16.7	0.003
		HILIC	Late Pregnancy	Preterm Birth	22.7	0.001

Galactose metabolism	4	C18	Late Pregnancy	Early Pregnancy – Late Pregnancy	13.2	0.031
		C18	Late Pregnancy	Preterm Birth	10.5	0.047
Glutamate metabolism	4	HILIC	Early Pregnancy	Preterm Birth	23.1	0.021
		HILIC	Late Pregnancy	Conception – Late Pregnancy	30.8	0.028
		HILIC	Late Pregnancy	Preterm Birth	23.1	0.019
		HILIC	Early Pregnancy	Conception – Early Pregnancy	23.1	0.040
Histidine metabolism	4	HILIC	Early Pregnancy	Preterm Birth	21.7	0.007
		HILIC	Late Pregnancy	Conception – Late Pregnancy	30.4	0.009
		HILIC	Late Pregnancy	Preterm Birth	17.4	0.030
		HILIC	Early Pregnancy	Conception – Early Pregnancy	21.7	0.016
Linoleic acid metabolism	4	HILIC	Early Pregnancy	Preterm Birth	18.2	0.026
		HILIC	Early Pregnancy	Conception – Early Pregnancy	27.3	0.003
N-Glycan biosynthesis	4	HILIC	Late Pregnancy	Conception – Late Pregnancy	33.3	0.011
		HILIC	Late Pregnancy	Early Pregnancy – Late Pregnancy	26.7	0.003
		HILIC	Late Pregnancy	Preterm Birth	20.0	0.032
N-Glycan degradation	4	C18	Early Pregnancy	Conception – Early Pregnancy	25.0	0.016
		C18	Early Pregnancy	Preterm Birth	25.0	0.036
		HILIC	Late Pregnancy	Early Pregnancy – Late Pregnancy	28.6	0.031
		HILIC	Late Pregnancy	Preterm Birth	28.6	0.037
TCA cycle	4	C18	Late Pregnancy	Conception – Late Pregnancy	23.8	0.006
		C18	Late Pregnancy	Preterm Birth	14.3	0.025
Branched-chain amino acid degradation	4	C18	Early Pregnancy	Conception – Early Pregnancy	15.4	0.012
		C18	Early Pregnancy	Preterm Birth	15.4	0.036
Vitamin B5 biosynthesis	4	C18	Late Pregnancy	Conception – Late Pregnancy	30.0	0.009
		C18	Late Pregnancy	Early Pregnancy – Late Pregnancy	30.0	0.003
		C18	Late Pregnancy	Preterm Birth	20.0	0.029
Methionine and cysteine metabolism	3	HILIC	Late Pregnancy	Early Pregnancy – Late Pregnancy	13.2	0.038
		HILIC	Late Pregnancy	Preterm Birth	15.1	0.021
Phytanic acid peroxisomal oxidation	3	C18	Early Pregnancy	Conception – Early Pregnancy	22.2	0.022
		C18	Early Pregnancy	Preterm Birth	22.2	0.049

Abbreviations: C18: reverse phase chromatography column, HILIC: hydrophilic interaction liquid chromatography column.

Table S5. Associations between ambient temperature during pregnancy and preterm birth in the Atlanta African American Maternal-Child Cohort, 2014 - 2020 (N = 215).

	Conception – Early Pregnancy		Early Pregnancy – Late Pregnancy		Conception – Late Pregnancy		Conception - Delivery	
	**OR (95% CI)**	**p-value**	**OR (95% CI)**	**p-value**	**OR (95% CI)**	**p-value**	**OR (95% CI)**	**p-value**
**Per 1 °C**	0.96 (0.85,1.08)	0.46	1.01 (0.90,1.14)	0.86	1.02 (0.87,1.19)	0.84	1.09 (0.83,1.44)	0.54
**Quartile 1**	*Ref.*	-	*Ref.*	-	*Ref.*	-	*Ref.*	-
**Quartile 2**	0.41 (0.09,1.96)	0.27	1.19 (0.29,4.93)	0.81	1.95 (0.48,7.91)	0.35	2.00 (0.80,5.03)	0.14
**Quartile 3**	0.54 (0.07,3.96)	0.55	0.34 (0.05,2.39)	0.28	0.92 (0.15,5.84)	0.93	2.11 (0.77,5.77)	0.14
**Quartile 4**	0.36 (0.04,2.90)	0.34	0.54 (0.07,3.95)	0.54	1.43 (0.20,10.33)	0.73	1.22 (0.40,3.96)	0.73

Abbreviations: OR: odds ratio, CI: confidence interval.

Note: Models adjusted for conception season, maternal age, education, body mass index, parity, use of alcohol, marijuana, or tobacco, gestational age at sample collection, and infant sex.

Table S6. Mean daily maximum ambient temperature (°C) for acute exposure periods during pregnancy, Atlanta African American Maternal-Child Cohort, 2014 - 2020 (N = 215).

Exposure Period	Mean (SD)
1-Week Before Early Pregnancy – Early Pregnancy	26 (7.7)
1-Week Before Late Pregnancy – Late Pregnancy	24 (8.4)

Table S7. Acute exposure period sensitivity analysis: Metabolic features enriched in the maternal metabolome, Atlanta African American Maternal-Child Cohort, 2014 - 2020 (N = 215).

Column	Metabolome	Exposure Period	p-value < 0.05	q-value < 0.05
HILIC	Early Pregnancy	1-Week before Early Pregnancy – Early Pregnancy	671	1
	Late Pregnancy	1-Week before Late Pregnancy – Late Pregnancy	946	0
C18	Early Pregnancy	1-Week before Early Pregnancy – Early Pregnancy	490	1
	Late Pregnancy	1-Week before Late Pregnancy – Late Pregnancy	496	0

Abbreviations: C18: reverse phase chromatography column, HILIC: hydrophilic interaction liquid chromatography column.

Note: Benjamini-Hochberg procedure used for multiple-test correction.

Table S8. Acute exposure period sensitivity analysis: Metabolic pathways enriched in the maternal metabolome, Atlanta African American Maternal-Child Cohort, 2014 - 2020 (N = 215).

Column	Metabolome	Exposure Period	p-value < 0.05
HILIC	Early Pregnancy	1-Week before Early Pregnancy – Early Pregnancy	12
	Late Pregnancy	1-Week before Late Pregnancy – Late Pregnancy	11
C18	Early Pregnancy	1-Week before Early Pregnancy – Early Pregnancy	15
	Late Pregnancy	1-Week before Late Pregnancy – Late Pregnancy	9

Abbreviations: C18: reverse phase chromatography column, HILIC: hydrophilic interaction liquid chromatography column.

Table S9. Metabolic features enriched in the maternal metabolome, Atlanta African American Maternal-Child Cohort, 2014 - 2020.

Column	Metabolome	Exposure Period	p-value < 0.05	q-value < 0.05
HILIC	Early Pregnancy	Conception – Early Pregnancy	662	0
		Preterm Birth	683	0
	Late Pregnancy	Early Pregnancy – Late Pregnancy	691	0
		Conception – Late Pregnancy	1103	13
		Preterm Birth	636	0
C18	Early Pregnancy	Conception – Early Pregnancy	484	0
		Preterm Birth	583	0
	Late Pregnancy	Early Pregnancy – Late Pregnancy	489	0
		Conception – Late Pregnancy	673	0
		Preterm Birth	535	0

Abbreviations: C18: reverse phase chromatography column, HILIC: hydrophilic interaction liquid chromatography column.

Note: Benjamini-Hochberg procedure used for multiple-test correction.
